# Working With Infertile Couples Seeking Assisted Reproduction: An Interpretative Phenomenological Study With Infertility Care Providers

**DOI:** 10.3389/fpsyg.2020.586873

**Published:** 2020-12-17

**Authors:** Federica Facchin, Daniela Leone, Giancarlo Tamanza, Mauro Costa, Patrizia Sulpizio, Elena Canzi, Elena Vegni

**Affiliations:** ^1^Department of Psychology, Università Cattolica del Sacro Cuore, Milan, Italy; ^2^Unit of Clinical Psychology, San Paolo University Hospital, Asst-Santi Paolo e Carlo, Milan, Italy; ^3^Reproductive Medicine Unit, Ospedale Evangelico Internazionale, Genoa, Italy; ^4^Reproductive Medicine Unit, San Paolo University Hospital, Asst-Santi Paolo e Carlo, Milan, Italy; ^5^Department of Health Sciences, University of Milan, Milan, Italy

**Keywords:** assisted reproductive technology, fertility team, infertility care providers, interpretative phenomenological analysis, lived experience, qualitative research

## Abstract

Although most studies investigated the impact of infertility and its treatment on the couple, a small body of evidence suggested that infertility care providers may experience different sources of stress related for instance to excessive workload, the complexity of the technique, and relational difficulties with patients. The current study aimed at providing further insight into the understanding of the subjective experience of infertility care providers by highlighting their feelings and emotions, personal meanings, challenges, and opportunities. Following the methodological guidelines of Interpretative Phenomenological Analysis, we conducted individual semi-structured interviews with 23 members of two different fertility units. Interviews were audiotaped and transcribed verbatim. Textual analysis was then conducted to identify emerging dominant themes and subthemes. Three main themes were extracted: (i) *dealing with infertile patients and their specificities*, (ii) *performing assisted reproductive technology (ART)*, (iii) *being part of a team*. These themes related to participants experiencing: (i) difficulties in establishing an empathic connection and communicating with couples, such that women were sometimes perceived as “particular patients” and men as poorly involved in the process; (ii) difficulties in dealing with a complex procedure in which errors are not allowed (as reported by embryologists), with a growing number of women aged > 40 seeking assisted reproduction, despite the risks for their health; (iii) being part of a team as a resource, although the huge amount of time spent together can involve conflicts and organizational problems. These findings suggested that patients’ overpersistence (rather than just dropout) represents an important source of stress for infertility care providers. At the same time, the concept of particular or difficult patient derives from the combination of multiple factors, including providers’ own history and subjective experience. The presence of mental health professionals in fertility units is essential to help providers improve the quality of doctor-patient communication and relieve the stress related to organizational issues and conflicts.

## Introduction

Nowadays, 9–15% of couples worldwide have difficulties conceiving (Boivin et al., 2007), and an increasing number of infertile couples have been seeking assisted reproductive technology (ART) to have a child (Laganà et al., 2017). According to the definition used by the Centers for Disease Control and Prevention (CDC), ART includes a variety of procedures aimed at treating infertility. All these procedures involve handling both eggs and embryos. In general, eggs are surgically removed from women’s ovaries, combined with sperm in the laboratory, and then reintroduced into women’s body or donated to another woman^1^.

Research has largely demonstrated that infertility and its treatment are associated with psychological distress, anxiety, and depression (Donarelli et al., 2016; Lakatos et al., 2017), as well as with sexual dysfunction, especially in women (Facchin et al., 2019). Couples undergoing ART experience a physically and psychologically demanding procedure, with low success rates (around 30% per cycle) (Ferraretti et al., 2013).

In the context of ART, dropout—which occurs when couples abandon treatment after a failed cycle, despite a favorable prognosis and absence of economic difficulties—depends on the complex interaction of patient factors (such as fear, negative attitudes to treatment, emotional, and relational strain), treatment factors (such as physical burden), and clinic factors related to organizational problems, as well as to difficult patient-provider interactions—see the interesting model, i.e., “Integrated Approach to Fertility Care”, presented by Boivin et al. (2012). Moreover, negative experiences of care are often mentioned by infertile patients as a reason for discontinuing treatment (Gameiro et al., 2012).

As regards this third set of factors, several studies have indicated that ART providers, and thus not only patients, have to cope with multiple sources of stress (Boivin et al., 2012), deriving for instance from organizational difficulties, with time pressure and work overload (Gerson et al., 2004; Klitzman, 2018). In a qualitative study by Simpson and Bor (2001), obstetric sonographers—who were interviewed to explore their experiences of giving bad news to women during ultrasound scans—reported that shortage of time, which did not allow for providing adequate support to patients and was associated with excessive workload, was perceived as stressful. On the other hand, less difficulties were experienced when a protocol providing clear indications on how to proceed following the communication of bad news was available in the workplace. In this regard, communicating with patients, which also involves dealing with their negative emotional reactions, especially in case of bad news, represents another significant source of stress for ART providers (Grill, 2015). As highlighted by Leone et al. (2017) in their qualitative study, these professionals may experience bad news as related to their own failure as clinicians, with feelings of disappointment, also associated with the fact that, in the context of ART, treatment success is still far from being guaranteed. In addition, the procedure is complex and involves high levels of responsibility, also considering the type of material (i.e., gametes and embryos) manipulated by ART professionals (Fitzgerald et al., 2013). These challenges may lead to frustration (and even to burnout) among providers, especially when the team is not able to guarantee the desired optimal standards of care (Grill, 2015).

This small body of qualitative research indicates that investigating the subjective experience of infertility treatment providers may be very important to improve professionals’ psychological conditions, with subsequent greater overall quality of care and patient satisfaction. However, this issue has been addressed by a small number of studies, and most research is still focused on the impact of ART on couples.

As suggested by the literature cited above (e.g., Simpson and Bor, 2001; Fitzgerald et al., 2013; Leone et al., 2017), qualitative methods can be particularly useful for researching the subjective experience of infertility care providers. Thus, we conducted the current qualitative study to explore in depth the characteristics of the lived experience of working in the context of ART as members of the clinic staff. Specifically, the shared meaningful experience explored in this study had two main components (i.e., being infertility care providers and being members of a fertility team), and our research question was: how do infertility care providers make sense of their experience of working in the context of ART as members of a fertility team? What are their feelings and emotions, perspectives and personal meanings, challenges and opportunities?

## Materials and Methods

In this article, our study is reported following the Standards for Reporting Qualitative Research (O’Brien et al., 2014; see, also, Hammarberg et al., 2016). The study was designed according to the theoretical and methodological principles of Interpretative Phenomenological Analysis (IPA) as described by Smith et al. (2009). IPA is a qualitative inductive approach aimed at providing in depth exploration of individuals’ lived experience, which also involves understanding personal meanings and perspectives (Smith and Osborn, 2015; Smith, 2019). IPA has been largely used in health research, especially in studies investigating patients’ subjective illness experience (see, for example, Smith et al., 2017; Larsson et al., 2019), but there are also IPA studies focused on caregivers (Hunt and Smith, 2004; Oliver et al., 2020), as well as on the lived experience of professionals working in stressful environments (Beryl et al., 2018; Volpato et al., 2018; Schaad et al., 2019).

We combined a sampling technique of convenience (such that we recruited those team members who were available when researchers were present) and purposive sampling to recruit participants of different professions, because we were interested in exploring the perspectives of all team members (gynecologists, biologists, midwives, nurses, and healthcare assistants). We did not apply any restriction regarding professionals’ nationality and age, or time since the beginning of their professional activity. Exclusion criteria were (1) not being able to understand and speak Italian and (2) not being a member of a fertility team (e.g., external collaborators of a fertility center). Following these criteria, final participants were 23 members of two fertility units recruited at two different public hospitals located in Northern Italy.

Ethical approval was obtained by the ethics commission of the Department of Psychology at the Catholic University of the Sacred Heart (Commissione Etica per la Ricerca in Psicologia; CERPS). Face to face semi-structured interviews were conducted in 2018 in a private room at the hospital by the first author and three young psychologists with an expertise in the area of ART. Written informed consent was provided by all participants, who received complete information regarding study objectives and procedures, including confidentiality protection strategies. Interviews were conducted using a storytelling approach, because we wanted our participants to narrate their personal experience as freely as possible. For this reason, each interview started with an open-ended question (“Could you start by telling me about your work experience in a fertility team?”) and continued in the form of a dialogue with questions aimed at exploring professionals’ lived experience in terms of feelings about their job, meanings, expectations, work challenges, and resources. Participants were also encouraged to disclose their personal ideas about ARTs. Field notes were taken by the interviewers. All interviews were tape recorded and subsequently transcribed verbatim. The duration of the interviews ranged between approximately 30 min and 1 h. All participants’ identifying details were omitted from transcriptions to protect confidentiality.

### Data Analysis

Textual analyses were conducted independently by two authors (FF, DL), but findings were constantly shared and discussed by the whole team throughout the analytic process (i.e., an iterative reflective process rather than a linear process). Consistently with the approach described by Smith et al. (2009), data analysis started with line-by-line reading of each interview with an exploratory attitude, and initial notes were taken to underline and summarize relevant topics, describe the language used by the participants, and provide preliminary interpretations when possible. The second step involved aggregating these initial codes to identify emergent themes for each participant (which moved the analytic process to a higher level of abstraction). When similar themes emerged from different interviews, we repeated the same theme title. In the third step, analyses were conducted across all participants looking for connections between the emergent themes identified in step two, which involved the creation of a conceptual map. Some of these themes were further clustered in superordinate concepts to capture the main components of participants’ lived experience. At the end of the process, we discussed our findings with the two fertility teams in two separate group meetings, and the feedbacks received by these professionals were used to improve our interpretation of the results and enhance the trustworthiness of our study. All discrepancies were discussed until full consensus was reached.

## Results

**FIGURE 1:**
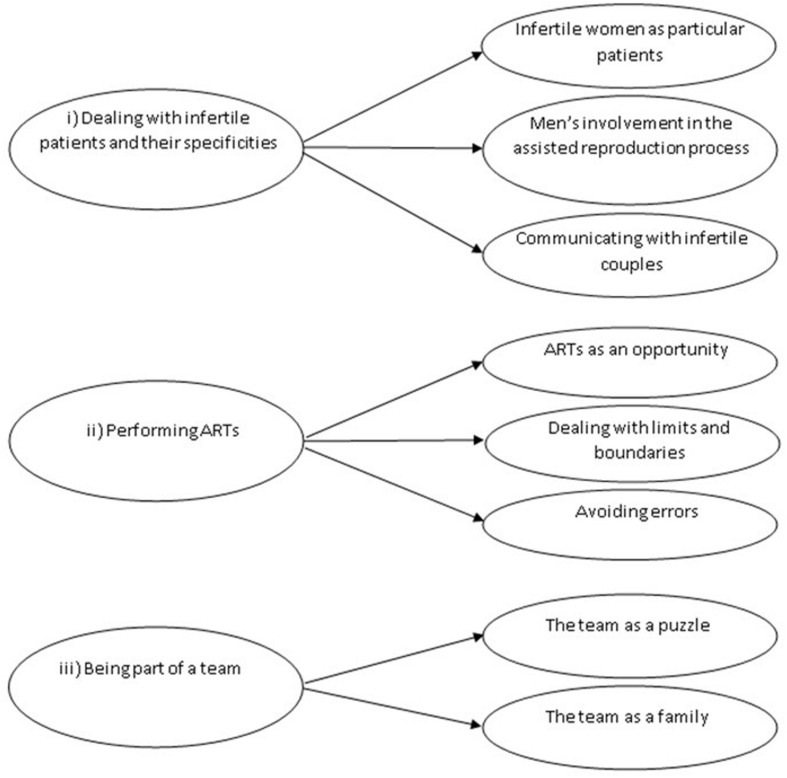
Dominant themes and subthemes extracted from textual analyses.

Twenty-five professionals were initially invited to participate in the study. All of them accepted our invitation, but 2 (a midwife and a psychologist) subsequently declined for lack of time. The sample was composed of 23 professionals [8 biologists and embryologists (35%), 5 gynecologists (22%), 5 nurses (22%), 4 healthcare assistants (17%), and one midwife (4%)]. Participants’ age ranged between 32 and 63 years (mean = 48.7; standard deviation = 7.9). Fifteen participants (65%) were married, 5 (22%) were in a relationship, and 3 (13%) were divorced. The majority of the interviewees [16 (69%)] had children (adopted, in one case). As a result of the analytic process described above, we identified 3 superordinate themes characterizing participants’ lived experience of working with infertile couples seeking ART: (i) *dealing with infertile patients and their specificities*, (ii) *performing ARTs*, (iii) *being part of a team*. These superordinate themes and their subthemes are represented in Figure 1.

### Dealing With Infertile Patients and Their Specificities

Most participants recounted difficulties working with infertile patients, and patient-related factors were described as a source of stress for providers, as well as an important obstacle in establishing a positive, empathic provider-patient relationship. This superordinate theme involved three subthemes that allowed to clarify how and why dealing with infertile couples was sometimes stressful for our interviewees. These subthemes were: (i) *infertile women as “particular patients”*; (ii) *men’s involvement in the assisted reproduction process*; (iii) *communicating with infertile couples*.

#### Infertile Women as “Particular Patients”

Infertile women overall—although with remarkable individual differences—were experienced as *“particular patients”* due to their intense feelings of anguish and depression, often translated into frustration, as well as into impatient demands, sometimes with a lack of trust in doctors and a tendency to blame them for unsuccessful treatments:

*“Infertile women are particular patients. They tend to be extremely anxious, worried; they feel like things will always go wrong for them; many women seem like they have the need to control everything”* (biologist).

Several participants used the words *“a child at all costs”* to describe these women’s particular *“need for a child”* (especially as regards older patients). On the one hand, all providers were fully aware of the psychological pain caused by infertility. Patients’ emotional labor was considered as an inevitable component of the IVF process, and all professionals tried their best to provide personalized, good quality care (which also entailed recommending psychological treatment, when necessary). On the other hand, it was difficult for them to deal with extreme situations, in which for instance severely distressed women claimed their *“right to have the belly”* despite multiple previous unsuccessful IVF cycles, with very limited chances of pregnancy (this situation was referred to as *“obsessive IVF”* by a nurse). These patients’ feelings and positions, combined with very high expectations and hostile attitudes, were difficult to understand by our participants, especially by those who recounted previous professional experiences in oncology units. These professionals (especially nurses) struggled to comprehend how and why such a great deal of psychological suffering could be related to the fact of having infertility, which is not a life-threatening condition:

*“I acknowledge the emotional burden, but no one is going to die, it’s not like in oncology units or intensive care”* (nurse).

Several interviewees stated that such intense feelings of distress might be due to cultural pressures (e.g., *“a woman must be a mother”*), as well as to painful comparisons with pregnant friends and in general with women who have been able to conceive:

*“Patients often say: why did she have her baby while I can’t?”* (gynecologist).

### Men’s Involvement in the Assisted Reproduction Process

This issue was raised by almost all participants, although with different positions. All professionals acknowledged that partners represent an important source of support for women throughout IVF. In this regard, a positive intimate relationship, characterized by good communication, sexuality, and care, was consistently identified as a fundamental factor that may significantly affect IVF psychological outcomes. Although most participants underlined the important role of partners and referred to IVF as a couple-centered process, a few providers described assisted reproduction as an unbalanced process, with women as protagonists in terms of decision-making, physical, and emotional involvement. These participants recounted situations in which men were completely absent, to the point of being defined as *“ghosts”* by an embryologist. In these situations, male partners’ involvement was experienced by the interviewees as a challenge in their relationship with the couple:

*“In general, I think men are less involved than women. […] 20% of male partners are on top of it, but 50% of them undergo the procedure like ghosts, leaving no traces. For other men, it seems like they are doing something unconceivable, a terrible effort. Then you remind them that their spouse is under anesthesia in the other room”* (embryologist).

*“Sometimes the husband is physically there, but mentally absent”* (nurse).

*“[…] I told her she had to come here with her husband, who had to sign the informed consent form. And she said, «My husband has to take three hours off work to come». I asked, «Is he going to be here with you on the transfer day?» and she answered, «No! I am coming with my mother!» […]”* (gynecologist).

When these findings were further discussed with both fertility teams at the end of the study, infertility etiology, and especially male factor infertility, was identified as an important variable associated with men’s lack of involvement. Participants hypothesized that a silent withdrawal may be the way in which men deal with negative feelings, such as shame about not being fertile, as well as embarrassment when providing semen samples.

### Communicating With Infertile Couples

For all the professionals in the study, a fundamental component of provider-patient communication was represented by pre-intervention counseling, whose aim was to guarantee comprehensiveness of treatment information (including rates of success, effects of pharmacological and surgical interventions, psychological implications of assisted reproduction, IVF-related risks, and causes of failure) and thus mentally prepare patients to possible negative results. However, this strategy was not considered as sufficiently effective in preventing patients’ overly high (or overly low) expectations, with negative psychological consequences in case of failure. This discrepancy was particularly challenging for providers, especially embryologists, who emphasized the importance of presenting technical aspects of IVF procedures and outcomes *(“the ratio of the technique,”* reporting the words of an embryologist). In case of unsuccessful interventions, embryologists can be required to provide very specific explanations regarding oocytes and embryos, which often occurs on the telephone. Detailing such a complex procedure to patients is extremely difficult and requires an accurate choice of type and number of words. The embryologists in the study acknowledged that working on language has been an important aspect of their professional growth:

*“I noticed that, when I started, I used to talk to patients as if I was giving a conference presentation. I think they were able to understand less than zero. Then I realized it would have been more functional to avoid technicism and thus use a simpler approach […]. Simplification made things easier, although I am still having difficulties explaining the procedure”* (embryologist).

### Theme 2: Performing ARTs

All participants perceived ARTs as an important opportunity to help couples become parents, but at the same time performing ARTs entails multiple stressful challenges and raises psychological and ethical issues that were discussed by our participants. This superordinate theme comprises three subthemes: (i) *ARTs as an opportunity*?; (ii) *dealing with limits and boundaries*; (iii) *avoiding errors*.

#### ARTs as an Opportunity?

Our participants described ARTs as a fundamental resource that may allow infertile individuals realize their dream of becoming parents, despite the presence of pathologies that would have been a definitive impediment 20 years ago. In this regard, patients’ happiness and satisfaction represented a major source of reward for providers:

*“I think ARTs give a great chance to infertile people, which has been revolutionary in our society. It is not comparable to lifesaving procedures such as transplants, but in some ways ARTs are mind-saving because you can touch these couples’ happiness when the child arrives”* (gynecologist).

Interestingly, several participants—especially those who had directly experienced infertility—expressed ambivalent feelings by saying that ARTs are indeed a great opportunity, but at the same time they would not seek assisted reproduction to have a child, for instance to avoid the negative consequences of the procedure on their intimate relationship:

*“The psychological burden of ARTs is huge […]. I am not sure I would seek ARTs in case of infertility problems […], I would probably prefer adoption. […] Based on my experience, there is a remarkable impact on the couple relationship […]. I would be worried about the relationship with my partner […]. For instance, sexuality may become a mechanical, unpleasant activity”* (embryologist).

#### Dealing With Limits and Boundaries

Although ARTs allow to overcome infertility, the low rates of success indicate that nature still sets boundaries of which our participants were fully aware. *“Nature can’t be pushed beyond a certain limit,”* claimed a biologist. Women’s age remains a major limit that should be clearly explained to patients:

*“I would never recommend ARTs to a 48-year-old woman, the risks for her health are very high. […] Let me give you an example. I received a phone call by a patient, whose 49-year-old sister in law underwent heterologous fertilization and got pregnant. She has now been hospitalized with severe hypertension, physicians are not able to treat it and thus suggested pregnancy interruption”* (gynecologist).

Consistently with these considerations, our participants underlined the importance of not perceiving themselves as *“creators of life”* in case of achieved pregnancy by the couple, or as *“failures”* after an unsuccessful cycle. The potential oscillation between these two positions was referred to as a dangerous psychological dynamic. Considering their own work as a small part of a more complex process was indicated as an effective protective strategy:

*“You really need to avoid getting too caught up in your feelings of guilt […], like, you know, I transferred the embryos and she’s not pregnant, it’s my fault […], but at the same time you can’t triumph when the woman shows up with the baby, as though you made it. […] This grandiosity is not appropriate. […] We are not failures and we are not creators of life. We are just well trained professionals who do their best together as small parts of the whole process that leads a couple to have a baby”* (embryologist).

#### Avoiding Errors

This subtheme specifically captured the subjective experience of embryologists, who discussed the importance of avoiding mistakes. These scientists highlighted the need for being constantly focused, which entails *“avoiding the theatre of emotions,”* as claimed by an embryologist. *“We don’t manipulate normal cells,”* stated another embryologist, *“each embryo represents a hypothetic future individual.”* Therefore, embryologists need to have *“a hundred eyes and a hundred hands.”* How to deal with this extremely high responsibility? Team work is very important: as reminded by almost all these participants, embryologists never operate alone. Some interviewees underlined the need for disconnecting from work at the end of the day by taking care of themselves and having a good time with their partner and children. Acknowledging that scientists are human beings with their own feelings and emotions was identified by a young embryologist as an important protective factor: being aware of one’s own limitations allows to ask for help and assistance when needed, rather than trying to make excessive efforts. This scientist quoted some Latin: “*Errare humanum est.*”

### Theme 3: Being Part of a Team

All participants, regardless of their function, perceived themselves as part of a group with specific dynamics, and the outcome of assisted reproduction was described as the product of a joint effort. The fertility team was described: (i) *as a puzzle* and (ii) *as a family*. Each representation led to specific consequences in terms of participants’ subjective experiences, resources, and challenges.

#### The Team as a Puzzle

Participants described the fertility team as a combination of differences, especially as regards members’ personalities. Like pieces of a puzzle, team members had complementary roles and personality traits. Such a combination was perceived as a fundamental resource in everyday practice:

*“Our team comprises multiple emotional worlds. We have the most anxious and the least anxious, the most courageous and the most prudent individual. This combination leads to a sort of mutual emotional correction”* (embryologist).

At the same time, dealing with diverse individuals, with different functions and work positions, was identified as a challenge and a potential source of organizational stress. For instance, the fact of having different types of contracts (which involved a different amount of work) was perceived as problematic in terms of work distribution.

#### The Team as a Family

*“I spend more time with my colleague than with my girlfriend,”* claimed a young biologist. Many other participants stated something similar while describing the significant amount of time spent at work with their colleagues. In this regard, the team was described as a family, and families have internal conflicts:

*“It becomes a sort of second family, or maybe even the first. Sometimes we fight, we may have conflicts”* (biologist).

## Discussion

To the best of our knowledge, this is one of the very few studies aimed at exploring the lived experience of working in a fertility team as reported by different professionals. Specifically, we used an IPA approach to explore in depth the characteristics of such an experience as narrated by 23 professionals working in two different fertility clinics. The themes and subthemes extracted led to the identification of sources of stress and vulnerability for professionals, as well as resources.

The first theme confirmed that infertile patients, and especially women, may be perceived as difficult due to their intense negative feelings of anxiety, pessimism, and frustration, such that communicating with these patients was experienced as particularly challenging by the professionals included in this study, as also reported by other authors (Fitzgerald et al., 2013; Grill, 2015; Boivin et al., 2017). For example, Fitzgerald et al. (2013) reported that embryologists can experience some patients as more difficult than others due to excessively high expectations, or simply due to being given inadequate information. In this regard, the embryologists included in our study underlined the importance of the quality of the information provided, suggesting that giving a great deal of technical details and statistics is not helpful, as previously underlined by Klitzman (2018). Therefore, our findings suggest that the type of information conveyed matters in the complex process of communicating with infertile patients, who are exposed to high levels of stress that may interfere with their understanding.

Our participants also addressed the importance of men’s involvement during treatment. In some cases, men were described as mentally and emotionally uninvolved, which seems to confirm the findings of Leone et al. (2018), who reported that in their study, focused on doctor-patient communication during ART visits, females talk accounted for 67% of overall patient talk. Taken together, these results highlight that the couple, rather than the woman, should be the real protagonist in the treatment of infertility.

Moreover, our findings offer further insight into the understanding of professionals’ difficulties with these patients by clarifying that providers’ own history and representations of infertility may hinder the development of an empathic doctor-patient connection. For instance, professionals with experience of cancer care may not fully understand the emotional burden of ARTs on infertile patients, because infertility is not a life-threatening disease. Therefore, as previously underlined by Grill (2015), the concept of “difficult patient” derives from the complex interaction of multiple factors that are not exclusively related to patient characteristics and behaviors.

Theme 2 explored interesting aspects related to participants’ perceptions of ARTs, with further indications regarding sources of difficulties and protective strategies used to relieve stress. The accounts revealed that the interviewees were fully aware of both the potential and the limits of the technique. Indeed, ARTs represent an important opportunity, but nature still sets boundaries and women’s advanced age remains an essential clinical issue. In fact, it is well known that women aged > 40 years seeking ARTs have high risks of health problems such as preeclampsia, gestational age, gestational diabetes, and preterm/very preterm delivery (Le Ray et al., 2012). As interestingly demonstrated by Klitzman (2016), the decision-making process in this situation entails dealing with a medical, psychological, and ethical dilemma, for instance regarding who decides and how the decision should be made. Our findings suggest that contemporary clinical practice with infertile patients seeking ARTs involves dealing not only with patients’ dropout, but also with overpersistence (which was referred to in our study as *“obsessive IVF”*), especially considering the increasing number of women aged 40 and above seeking ARTs (Klitzman, 2016).

On the other hand, fear of making mistakes has been acknowledged as a major source of stress by the embryologists in the study, who highlighted the importance of taking care of themselves by disconnecting from work and enjoying some time with their loved ones. In this regard, the embryologists included in a study by Fitzgerald et al. (2013) also emphasized the importance of avoiding errors with such irreplaceable material and discussed the importance of care of the self, which is not that common among other categories of health care workers. In our study, considering themselves as part of a more complex process, as an alternative to an individualistic approach, was described as another protective strategy.

In this regard, our study also demonstrated the importance of the team, which was perceived as a source of stress and a protective factor, at the same time. On the one hand, dealing with individual differences (also related to work functions) could be tiresome and generate conflicts, especially if one considers the significant amount of time that the team members spend together. The fact that organization and team dynamics may cause stress in fertility care providers has been underlined by other authors (Fitzgerald et al., 2013; Boivin et al., 2017). On the other hand, our findings also revealed that working in a group composed of people with different personalities can be helpful, since it facilitates the management of everyday stress, especially among embryologists.

The positive aspects of our study are related to the methodology used, which allowed for in-depth exploration of the participants’ experience, and to the novelty value of our findings, especially considering the paucity of research on this neglected topic. However, the generalizability of these findings is scarce, which should be acknowledged as a limitation. In fact, consistent with the IPA methodological guidelines, our sample was small (although quite large for an IPA study) and did not allow for systematic comparisons between different professional categories (e.g., gynecologists vs. embryologists), also considering the influence of other variables, such as participants’ age and years of experience in a fertility unit.

Because of these limitations, our results can open new research questions, rather than lead to firm conclusions. For instance, the individual, relational, socio-cultural and environmental factors that may lead to the concept of “difficult patient” in the context of ARTs require further investigation: there is need to clarify how and why some patients are perceived as more difficult than others, which would be very useful for clinical practice. Moreover, patients’ overpersistence—rather than just dropout—deserves further attention in order to identify the psychological processes and sociocultural influences underlying this complex mechanism. In addition, investigating doctor-patient communication in the context of infertility remains essential.

Our findings also have interesting clinical implications, since they underline the importance of mental health professionals in fertility units, not only to support patients, but also to work with fertility care providers. As also acknowledged by other authors (e.g., Grill, 2015), mental health professionals have the responsibility to help fertility care providers manage “difficult patients” and improve their capacity of establishing an empathic connection with them. In this regard, mental health professionals can work with providers to enhance their communication skills, as well as their understanding of the negative feelings related to infertility (fear, anguish, frustration, sense of inadequacy) underneath patients’ expressions of anger, lack of trust, and controlling behaviors (Patel et al., 2018). As suggested by Smorti and Smorti (2013), psychologists may also help providers understand more in depth the pathways to parenthood of couples who underwent ART, considering the specificities of this transition in the context of infertility (for instance, as regards to challenges and obstacles, sense of victory when the pregnancy is achieved, medicalization, and controlling behaviors). Moreover, psychologists can help providers understand whether their own history and subjective experience interferes with their clinical practice, especially in terms of doctor-patient communication. Mental health professionals can also provide useful interventions in case of work stress related to team dynamics, which may help providers better understand and avoid the negative group mechanisms that lead to tension, with improved ability to manage conflicts.

## Conclusion

In conclusion, national health care policies in the context of infertility should consider the findings provided by the small body of literature focused on fertility care providers to further enhance the presence of mental health professionals in the fertility staff.

## Data Availability Statement

The full texts of the interviews and participant information cannot be publicly shared due to privacy and ethical restrictions. Requests to access the study data should be directed to federica.facchin@unicatt.it.

## Ethics Statement

This study was reviewed and approved by the Commissione Etica per la Ricerca in Psicologia (CERPS), Department of Psychology, Catholic University of the Sacred Heart. The patients/participants provided their written informed consent to participate in this study.

## Author Contributions

FF, DL, GT, EC, and EV conceptualized the study. EV was also the supervisor. FF and DL analyzed the data. FF, DL, GT, and EC wrote the first draft of the manuscript, with suggestions from all authors. MC and PS supervised the findings of this study and edited the final version of the manuscript. All authors discussed the results and commented on the manuscript.

## Conflict of Interest

The authors declare that the research was conducted in the absence of any commercial or financial relationships that could be construed as a potential conflict of interest.

## Footnotes

1 www.cdc.gov/art/whatis.html
